# Perceptions of intermittent preventive treatment of malaria in pregnancy (IPTp) and barriers to adherence in Nasarawa and Cross River States in Nigeria

**DOI:** 10.1186/1475-2875-12-342

**Published:** 2013-09-23

**Authors:** Chamberlain C Diala, Thaddeus Pennas, Celeste Marin, Kassahun A Belay

**Affiliations:** 1 FHI360, C-Change Project, Global Health Population and Nutrition Division, 1825 Connecticut Avenue, Washington DC, NW 20009, USA; 2 New Haven, CT, USA; 3 US Agency for International Development (USAID), Abuja, Nigeria

**Keywords:** Malaria in pregnancy, Intermittent preventive treatment in pregnancy, Sulphadoxine-pyrimethamine, Antenatal care, Focus group discussions, In-depth individual interviews, Cross River State, Nasarawa State

## Abstract

**Background:**

Malaria during pregnancy is dangerous to both mother and foetus. Intermittent preventive treatment of malaria in pregnancy (IPTp) is a strategy where pregnant women in malaria-endemic countries receive full doses of sulphadoxine-pyrimethamine (SP), whether or not they have malaria. The Nigerian government adopted IPTp as a national strategy in 2005; however, major gaps affecting perception, uptake, adherence, and scale-up remain.

**Methods:**

A cross-sectional study was conducted in peri-urban and rural communities in Nasarawa and Cross River States in Nigeria. Study instruments were based on the socio-ecological model and its multiple levels of influences, taking into account individual, community, societal, and environmental contexts of behaviour and social change. Women of reproductive age, their front-line care providers, and (in Nasarawa only) their spouses participated in focus group discussions and in-depth individual interviews. Facility sampling was purposive to include tertiary, secondary and primary health facilities.

**Results:**

The study found that systems-based challenges (stockouts; lack of provider knowledge of IPTp protocols) coupled with individual women’s beliefs and lack of understanding of IPT contribute to low uptake and adherence. Many pregnant women are reluctant to seek care for an illness they do not have. Those with malaria often prefer to self-medicate through drug shops or herbs, though those who seek clinic-based treatment trust their providers and willingly accept medicine prescribed.

**Conclusions:**

Failing to deliver complete IPTp to women attending antenatal care is a missed opportunity. While many obstacles are structural, programmes can target women, their communities and the health environment with specific interventions to increase IPTp uptake and adherence.

## Background

Malaria in pregnancy (MiP) is a major health concern in Nigeria. Malaria infection is more dangerous during pregnancy, and adverse effects are more serious for the pregnant woman as well as the foetus and newborn. In endemic areas such as Nigeria, women have high levels of immunity so may not experience fever or other malaria symptoms. During pregnancy, however, their immunity is altered and they are more vulnerable to complicated and severe malaria. Meta-analysis of intervention trials in sub-Saharan Africa [1] suggests that in endemic areas, MiP is largely undetected and untreated, and leads to 100,000 infant deaths per year due to malaria-associated, low birth weight. Malaria-related anaemia may cause up to 10,000 maternal deaths per year [2]. Malaria also increases high blood pressure in babies [1,3-5]. In Nigeria, a large proportion of pregnant women do not go to a health facility even when they have malaria symptoms. This is especially true in States where many Muslim women are in seclusion and do not make their own decisions about attending a health facility.

Intermittent preventive treatment of malaria in pregnancy (IPTp) is a strategy where all pregnant women are given a full curative dose of sulphadoxine-pyrimethamine (SP) at least twice during pregnancy, regardless of whether they have malaria. Starting as early as possible in the second trimester, IPTp-SP is recommended by the World Health Organization (WHO) [6] for all pregnant women at each scheduled antenatal care (ANC) visit until the time of delivery, provided that the doses are given at least one month apart. Sulphadoxine-pyrimethamine should not be given during the first trimester of pregnancy; however, the last dose of IPTp-SP can be administered up to the time of delivery without safety concerns. The effectiveness of IPTp-SP in improving birth weight and reducing prevalence of pre-term deliveries and maternal anaemia in Nigeria has been documented [1]. IPTp is part of Nigeria’s three-pronged approach to MiP: 1) prompt and effective case management of malaria; 2) use of IPTp with at least two doses of SP; and, 3) consistent and correct use of long-lasting insecticide-treated nets (LLINs).

Nigeria adopted IPTp as a national strategy in 2005, replacing weekly prophylaxis [7-9]. IPTp with SP should be offered as part of focused ANC. Focused ANC is a WHO-recommended approach that consists of a minimum of four goal-oriented ANC visits during a pregnancy. Focused ANC is part of the Federal Ministry of Health (FMoH) policy, but has not yet been scaled-up in most of the country. National protocols stipulate that SP is given free of charge through ANC services at public health facilities and non-governmental organization (NGO) facilities, using directly observed therapy (DOT).

The Government of Nigeria, development partners, and funding organizations have initiated several strategies and programmes to mitigate the impact of malaria on pregnant women and their children, but Nigeria has a long way to go in achieving targets set for IPTp. Recent studies revealed that few pregnant women adhere to the recommended two-dose course [10-12]. The 2010 Nigeria Malaria Indicator Survey reported that only 15% of women who had given birth in the two years preceding the survey had received even one dose of SP during their ANC visits, less than a third of the number who attended ANC with a skilled provider.

A number of quantitative studies have documented low level of IPTp adherence and identified knowledge gaps [13-16], but little is known about social and cultural barriers operating at individual, community and health facility levels that influence IPTp uptake and adherence among pregnant women. This study was designed to gather evidence on real and perceived barriers to IPTp-SP adherence from the perspectives of pregnant women and ANC providers.

### Objectives

The study objectives were to identify pregnant women’s and providers’ perceptions of IPTp, and barriers to adherence to the protocol of at least two doses. The objective of focus groups with women was to understand:

a) Real and perceived logistical, social and cultural barriers to correct IPTp use/adherence

b) Women’s ability to act and access appropriate care

c) Perceptions of risk of MiP

The objective of provider interviews was to understand:

d) Provider knowledge of MiP and IPTp

e) Perceptions of their role in ensuring adherence to IPTp

f) Institutional-level barriers

Qualitative data from pregnant and post-partum women and front-line ANC providers in tertiary, secondary and primary health facilities can not only help to fill knowledge gaps, but also inform and support the development of strategies and interventions that improve IPTp uptake and adherence.

## Methods

### Setting and population

Cross River and Nasarawa States have very different climatic, cultural and socio-economic indicators, but they share similar demographic and health indicators and a high malaria burden. As Table 1 shows, uptake of maternal health care is higher in Cross River than in Nasarawa State, and IPTp uptake in both states is higher than the national average, but still very low [17]. In 2010, the proportion of Nigerian women who received ANC from a skilled provider was estimated at 58% (66% in the North Central region, where Nassarawa is located and 74% in South-South, where Cross River is located) [17]. Table 1 describes the demographic and health indicators for study respondents and contrasts national with state-specific health indicators.

**Table 1:** Demographic and health indicators

**Indicator**	**National**	**South-South Region (Cross River)**	**North Central Region (Nasarawa)**
Women without formal education*	39.9%	14.9%	39.3%
Mean number of children ever born per woman*	6.4	6.2	6.4
Under-five mortality (per 1,000 live births)*	171	138	135
At least one ANC visit with a skilled health professional**	57.5%	73.6%	66.3%
At least one dose of IPTp at ANC**	14.6%	22.2%	7.1%
Two doses of IPTp at ANC**	13.2%	20.4%	7.1%

*Source: National Population Commission and ICF Macro 2009; ** Source: National Population Commission and ICF Macro 2011.

### Study design

**Figure 1:**
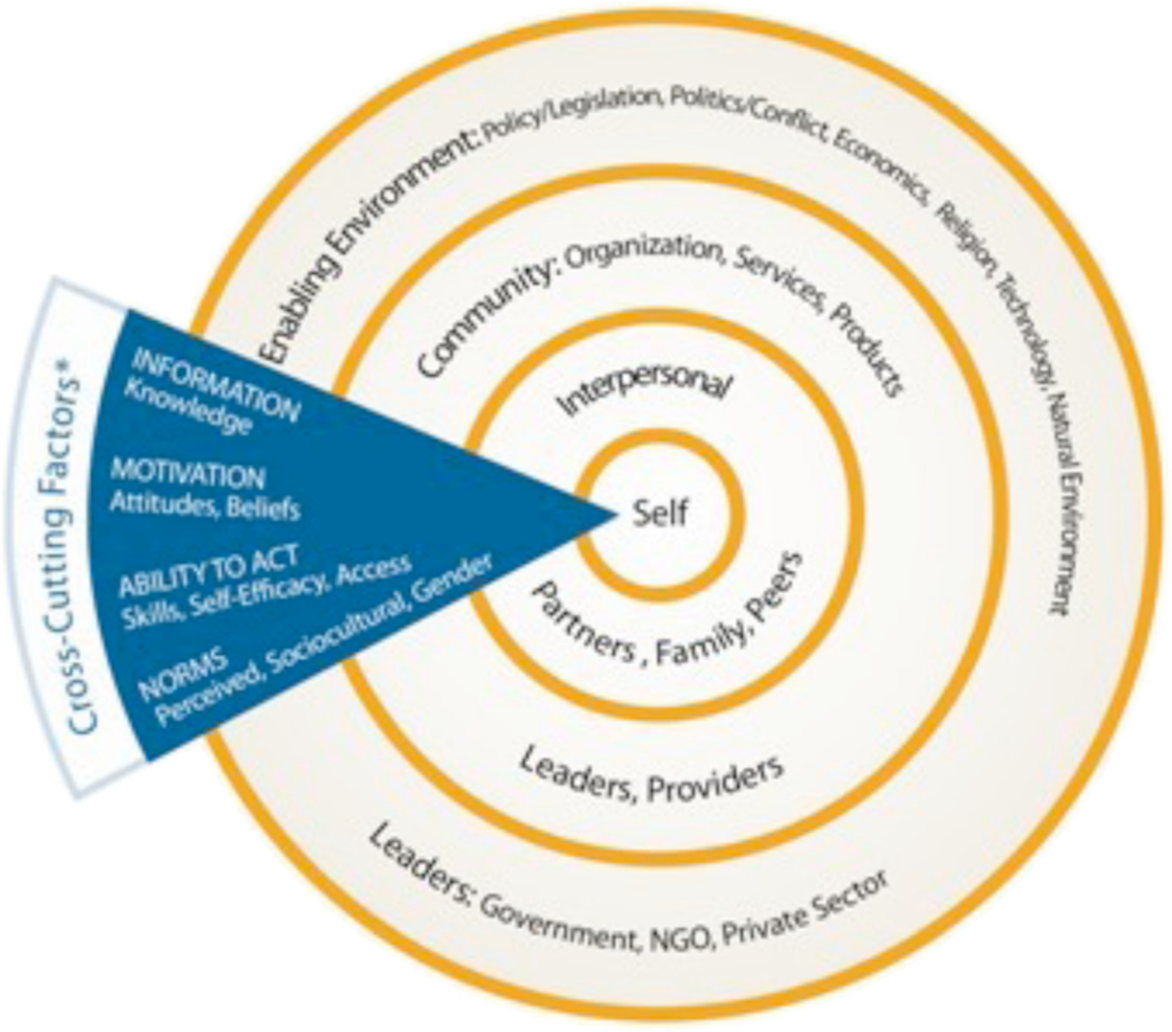
The socio-ecological model is adapted as the conceptual framework for this study.

The cross-sectional study was conducted in peri-urban and rural communities in Cross River and Nasarawa States in July and August 2012. Study instruments were based on a socio-ecological model and the multiple levels that influence or present obstacles to change are shown in Figure 1[18]. The model takes into account the individual, community, societal, and environmental contexts of behaviour and social change, including cultural norms, traditions and gender roles personal, societal, and religious beliefs; and, systemic, institutional and environmental factors.

Focus group discussions (FGDs) were conducted with women aged 20–40 years who were pregnant, post-partum, or had given birth in the previous two years; adolescents aged 14–19 years who accessed ANC and given birth in the previous two years; and in Nasarawa State, a “men-only” FGD was conducted with husbands of women who fitted the recruitment criteria and had also accompanied their wives to ANC facilities.

Adolescents were included as a separate group because they often have age-specific concerns about health care, and stigma regarding teenage pregnancy may prevent them from seeking health care at government facilities. It was important to gather the views of male partners in a State such as Nasarawa, which has a large Muslim population, in light of the dominant role of men in women’s use of health facilities [19].

In-depth interviews (IDIs) were conducted with community-based and facility-based health care providers who offer routine ANC and treat pregnant women with malaria. Interviews with health care providers were designed to elicit the following information:

● knowledge of how malaria affects pregnant women and malaria signs and symptoms

● whether treatment for malaria during pregnancy is a part of focused ANC services

● detailed knowledge of IPTp, including the names of medicines, dosages, availability, and cost

● evidence on institutional-level barriers to adherence, including stock-outs and insufficient training

● incentives for pregnant women to return for the second IPTp-SP dose

### Sampling and data collection

Ten study facilities that provide ANC services were purposively selected in each State: one tertiary facility, three secondary facilities and six primary health facilities. Fieldwork took place in two local government areas (LGAs) in Cross River (Calabar and Yakurr) and in three LGAs in Nasarawa (Keffi, Karu and Nasarawa Eggon). After a flood damaged one of the selected primary health facilities in Nasarawa, it was replaced by a district hospital, bringing the number of secondary facilities in Nasarawa to four.

Three interviews were conducted in each facility with physicians, nurses, midwives or community health extension workers who deal directly with pregnant and post-partum women. The number of FGDs depended on participant availability (Table 2). Most focus group discussions were conducted in available meeting rooms in the selected health facilities. Other FGDs were held in community centres, recreation halls, and schools.

**Table 2:** Focus group discussions in Cross River State and Nasarawa State

		**Cross River**		**Nasarawa**	
		**FGDs**	**Participants**	**FGDs**	**Participants**
Pregnant women aged 20–40 years who attend an ANC facility	Rural	3	30	3	30
	Peri-urban	2	20	2	20
Post-partum women aged 20–40 years who have attended an antenatal facility	Rural	3	30	3	30
	Peri-urban	2	20	2	20
Women aged 20–40 years who gave birth in previous two years and attended an ANC facility	Rural	3	30	3	30
	Peri-urban	2	20	2	20
Adolescent women (14–19 years of age) who have given birth and attended an ANC facility	Peri-urban	2	20	2	20
Husbands (men only) of pregnant or post-partum women aged 17–35 who gave birth in previous two years	Rural	--	--	1	10
	Peri-urban	--	--	1	10
**TOTAL**		**17**	**170**	**19**	**190**

Table 2 describes the characteristics of the study population and the number and location of FGD conducted with each group.

ANC clients at the selected facilities who met the age criteria were recruited to participate in the FGDs. Husbands of women who met study criteria were recruited into two “men only” FGDs in (rural and peri-urban) Nasarawa State. Initially, the study recruited women who had accessed ANC services because that is where IPTp is most often provided. The study was later expanded to include women who had not received ANC because large numbers of women were not attending ANC. These participants were recruited from communities; local leaders and chiefs assisted with recruitment, using town criers to announce the study and invite participation.

FGD facilitators were of the same gender as participants and spoke the same languages. Field workers attended a two-day training that covered subject selection and recruitment, instruments and techniques, facilitation and note-taking skills. The pre-tested discussion guide and other survey instruments were translated into local languages: Efik, Lokarr and Pidgin in Cross River and Hausa in Nasarawa, then back-translated to English.

Just before the FGDs, a short questionnaire covering basic demographic information including occupation, estimated household income, marital status, number of pregnancies and attendance at ANC was administered to about half of the participants.

### Conceptual framework and organization of findings

Audiotaped FGD sessions were transcribed and translated into English in the field to enable verification and first-level corrections. The data were then cleaned and imported into Atlas ti software for analysis. Data were coded according to a framework based on the conceptual model in Figure 1. Additional codes were created for themes that emerged from the data and transcripts were re-coded. Figure 1 is a modified socio-ecological model (SEM) with cross-cutting factors that identify the three levels (individual, community and environment) that impact women’s ability to make healthcare-seeking decisions.

Modifying the SEM for change, the study examined factors that may motivate or impede the ability of pregnant women to act promptly and decisively to prevent and treat malaria at three levels (individual, community and environment).

1. Individual: factors related to individual personality and autonomy, including beliefs about malaria and the efficacy of modern medications. This includes women’s knowledge of MiP and understanding of IPTp. Women do not act alone, but are also heavily influenced by their families: husbands, significant others, mothers-in-law and other social circles play a role in women’s access to care. In the conceptual framework, the individual level consists of the woman (the centre of the SEM) and her immediate family, particularly her partner, from the “interpersonal” level of the model.

2. Community: The framework further expanded the interpersonal to include other community level factors that encompass the influence of in-laws, preferences for home births, preferences for traditional birth attendants and other relatives, friends, neighbours, peers and others in the community, those in the “interpersonal” level of the model but outside the immediate family unit. Other community factors relate to the culture and norms of the community, for example with regard to whether pregnant women are expected to seek care in health facilities, with traditional providers, or a combination of the two. Issues of spirituality and witchcraft play important roles in women’s beliefs and actions.

3. Environment: The third framework level is the “environment” as most factors are institutional or systemic and the term “environment” encompassed the two outer circles of the model. From the women’s perspective, this includes economic factors, women’s willingness to follow provider advice, and systemic factors at ANC facilities, such as waiting times and attitudes of providers. From the providers’ perspective, this includes MiP knowledge, training on MiP and focused ANC, and stock-outs. In addition, poor attitudes, a lack of professional commitment for quality and inappropriate charges for hospital forms on the part of providers hinder women’s abilities to seek and adhere to ANC services.

Factors at the first two levels — individual and community — primarily relate to decisions to seek care (both routine ANC where IPTp should be provided and treatment for malaria if needed), accessing care at facilities and complying with provider instructions. Decisions to seek care are influenced by an understanding of MiP and its risks, perceptions of the effectiveness and quality of care, and support from family and community. Family and community support also influences women’s ability or motivation to access services and comply with instructions and adhere to medication regimen. Many factors at the environmental or institutional level relate to the kind of care pregnant women receive and are more specific to whether and how IPTp is provided.

## Ethical considerations

The Federal Ministry of Health’s National Health Research Ethics Committee provided ethical clearance for the study. Data collectors for this study successfully completed the Nigerian Ethical Training component of the Collaborative Institutional Training Initiative (CITI) on Human Subjects Research. All participants and parents or guardians of participants under age 18 provided written informed consent in English or their local language before joining a FGD. Those who could not read signed with a thumbprint.

## Results

### Demographic background and uptake of antenatal care

Most focus group participants who completed the pre-discussion demographic survey (N = 150) were currently or previously married, had at least a primary education and lived in a household with at least one income earner. Percentages by State are shown in Table 3. Nine per cent of the Cross River sample and 24% of the Nasarawa sample reported they were housewives. Table 3 describes the demographic characteristics of the study participants, including marital status, education and family income. In this study, family income was determined by three questions (Table 3) that explored respondents’ level of comfort with what they consider “current family income” since women in Nigeria simultaneously earn formal (specific occupations) and informal incomes (selling or trading goods and services). As a result, women are unable to provide specific amounts for current income due to changing seasons or variations in the products, goods and services they sell at any given time in the year.

**Table 3:** Demographic characteristics of focus group participants (N = 150)

	**Cross river**	**Nasarawa**
	**%**	**%**
**Marital status**		
Married	82	93
Single, never married	13	8
Divorced, separated, widowed	5	0
**Education**		
None	1	22
Primary	38	23
Secondary or higher	61	55
**Family Income**		
Live comfortably on current income	52	42
Getting by on current income	10	17
Find it difficult/very difficult to live on current income	36	39

As expected from the sampling approach, most participants had been to an ANC facility during pregnancy, though a smaller proportion in the group under age 20 had done so. (Some participants received ANC from non-facility-based community health workers only.) In both States, a majority of focus group respondents reported making four or more ANC visits; some in Nasarawa made over eight visits. Participants tended to describe ANC as services to protect the health of the foetus more than the health of the pregnant woman. Women described such ANC services as blood pressure and haemoglobin checks, palpation, family planning, immunization, personal hygiene and nutrition, but only rarely malaria.

Reasons that participants gave for some women not attending ANC included beliefs that only the seriously ill go to a health facility, that pregnancy and delivery are natural and do not require hospital care, and that hospital care offers unwelcome exposure to complications as well as unwelcome advice:

“If you advise them to go for antenatal, they refuse; they prefer to go to hospital only when pregnancy is due”.

“Some don’t want to come because they think coming to the clinic will cause complication and some say because they talk to them about family planning and other things like that”.

## First level of influence: individual

### Perceptions of malaria and prevention methods

FGD participants were asked what causes malaria and how it is transmitted, since such degrees of perceptions often determine what steps are taken to prevent illness and decisions to seek care [20,21]. In both States, most participants referred to mosquito transmission and cited appropriate signs and symptoms, such as headache, fever, lack of appetite, weakness of joints, and vomiting.

Participants also reported additional causes of malaria. A few noted that malaria may result from an attack (“witchcraft”) by somebody. More frequently, participants linked malaria to a virus, exposure to the sun, infection from toilets, or drinking bad (impure) water and connected the presence of mosquitoes to unclean or dirty environments:

“Leaving dirty things around the house causes malaria; leaving the toilet dirty, not washing and leaving dirty water around the house can cause malaria. Leaving unwashed plates or clothes can cause malaria; some people don’t tidy up their rooms”.

With the exception of one FGD in Cross River, all participants could name one or two proven malaria prevention methods such as use of LLINs, though use of LLINs was not mentioned in four of the 36 FGD sessions. In both States, participants cited insecticide spray and screened environments. No respondent mentioned indoor residual spraying.

Many participants believed in prevention methods that were not related to mosquitoes, such as washing hands after toilet use, cutting grass, staying out of the sun, and keeping premises clean. To repel mosquitoes, participants mentioned using a local rat poison called *otapiapia* and burning fragrant or “scenting” leaves:

“I keep our environment clean, because sometimes it’s not even about the nets. You may sit outside resting and the mosquito will come and bite you. You keep your compound clean and all the grasses around you, you clear it, so that mosquito will not come to your compound because it is the neat one. When you go to toilet, you wash your hands and you keep your toilet clean. That will prevent you not to have malaria”.

### Perceptions of risk and care-seeking for MiP

While some women could not identify any danger to the foetus from MiP, most perceived dangers such as anaemia, jaundice, miscarriage, pre-term birth, and stunted growth. Some participants referred to malaria as a cause of death and to community perceptions of its dangers.

“Malaria kills faster than AIDS, so most of them are scared”.

“[The foetus] could die inside [the mother]. Because of the high temperature, the foetus will try to get out [and the mother] could suffer a miscarriage”.

When asked about their motivation for visiting a government health facility for pregnancy-related care, most participants mentioned the need to maintain their health and particularly that of their unborn babies. Some said that going to a government health facility was their way of ensuring their baby’s safety. As one rural FGD participant put it,

“If we don’t have transport, we often walk to the health facility, no matter how far away we live”.

Most FGD participants said that if they had malaria, they would go to a hospital and take prescribed medications, if they could afford it. Nearly all participants said they would advise a pregnant woman to go to a health centre for malaria medication and indicated they know women or girls who have had malaria. Other barriers to accessing MiP care from health facilities mentioned included individual choice, ignorance, lack of money, lack of time, fear of surgery, dislike for oral medication, and reliance on prayer.

Traditional therapy was also highly acknowledged as a good cure for MiP. Many participants confirmed that seeking treatment in modern health facilities is generally viewed as a last resort, usually when the disease poses a major threat to life. A few participants reported that some traditional healers warned against mixing traditional and hospital medicines, and they admonished pregnant clients not go to a hospital for preventive care. Local herbs in Cross River, called *okon-a-tekor* and *yabulikponben,* are used to treat malaria, along with lemon grass and the leaves of *dongoyaro* (Neem), pawpaw, mango, and lime trees. Advantages that participants cited were that traditional medicine works faster and is cheaper and more conveniently accessed, in comparison to anti-malarial medicines that smelled bad, caused nausea, side effects and allergic reactions, and were “very big to swallow”.

“My friend here dislikes oral medicine, so when she collects the medicine she will hide it under a pillow and later throw it away”.

“Some if they take it (anti-malarial drugs) they vomit, so they don’t take it”.

Side effects of modern anti-malarials mentioned tended to be transient, affecting the woman, rather than potential harm to the foetus. A few participants noted that traditional herbs involved unreliable dosages and regimens. Respondents also referred to the potential danger of patronizing “chemists” instead of getting approved anti-malarial medicines from government health facilities.

### Understanding of IPTp

Some participants perceived malaria prevention as a component of ANC, but most did not. Very few participants in rural areas referred to receiving anti-malarial drugs, LLINs, or rapid diagnostic testing of malaria with ANC services. Participants did not appear to clearly understand the difference between chemoprophylaxis (prevention of malaria through medication) and the specific treatment they have to take in the event of an episode of malaria. In nearly all FGDs, participants correctly identified the appropriate anti-malarial drugs used during pregnancy, including the SP brand names Laridox and AntiMal. Some participants mentioned chloroquine, which was frequently used as prophylaxis in the past, but has since been withdrawn due to resistance. All participants in one FGD in Cross River identified Coartem rather than SP as the drug of choice for pregnant women. Most women trusted their health facilities and said they attempted to follow instructions given to them by the doctors, nurses or midwives regarding the dosage of malaria medication, but the dosage and the spacing of the two doses are not clearly understood.

Some participants referred to the need to take two doses while pregnant, but the majority of women in nearly all the FGD sessions demonstrated inadequate knowledge, such as incorrectly describing the dosage of SP or the recommended interval between doses. Only a few women knew how many tablets constituted the recommended dose or that the requisite number of tablets were to be swallowed at the same time under the direct observation of a health provider (DOT).

“I wanted to take the three tablets at once but somebody told me that I should not take the three tablets at once, that I should take one per day, so that was how I took my own”

“They instruct us to take the medicine in the morning, afternoon and in the evening, every day for the number of days they will tell us”.

“Well, we do not know when to take this medicine. Whenever we come to the clinic and they prescribed medicine for us they only tell us to take 3 times or 2 times. Since we have never been diagnosed with malaria, we don’t really know when”.

“The women who are registered here are given the drugs upon registration and again when they are six months pregnant, so that you are supposed to take two doses before childbirth”.

### Family support for seeking MiP care

Community or social factors affecting women’s health-seeking behaviour include the support they receive from husbands, friends and other relatives. Male partners’ influence on the uptake of maternal health services has been well documented [22]. Men play an important economic role, including paying for transportation to and from health facilities, hospital services, prescriptions, and recommended foods.

### Husband’s role – from women’s perspective

Fewer women under 20 years acknowledged the importance of husbands’ support, but women over 20 years in both States referred to it frequently.

“Our husbands are very supportive if they notice any sign of malaria in pregnancy. They are aware that we can only get good treatment in hospitals, and they will not be able to concentrate on other things if our health is in trouble. He will always encourage me to go to the hospital to confirm the health of both the baby and the mother. They always support us with money for treatment so that we can give anytime such is required”.

FGDs with both women and men suggested that a pregnant woman has no alternative if her husband wants her to seek care in a health facility. One of the main types of support husbands provide is money for services and transport.

“Sometimes they offer advice; sometimes they offer support by giving transport money”.

Beyond paying for services, some women said their husbands encouraged them to take their medications, reminded them of scheduled visits, and monitored their health. Some husbands accompany their wives to the ANC facilities, and others asked a female relative (often a sister) to accompany their wives.

More FGDs in Nasarawa reported that husbands were very supportive of them seeking modern, medical care for MiP; only a few reported otherwise. Views from Cross River were more varied, ranging from support to ambivalence, indifference or hostility toward modern medicine. In Cross River and Nasarawa States, poverty or lack of money was considered the main, but not the only reason some men did not encourage their wives to go to the health facility.

Religion and traditional beliefs were considered barriers for some in both States. In Cross River, a preference for traditional healers was cited, along with the belief in adhering to practices of parents and older generations who did not go to ANC facilities.

“Some, like my husband's tribe, dislike medicine; most of them do not like hospital. When they are sick, they patronize [a traditional medicine practitioner]. I had an experience three months ago and I was bleeding during pregnancy, my husband and his family took me [there]”.

“They (some husbands) are very greedy, they don’t want to spend money, they say the traditional way is faster and refuse to bring the money to allow them to come”.

Only rarely was sickness in pregnancy described as being caused by witchcraft and therefore only curable by traditional medicine. More often, traditional medicine consisted of herbal remedies or tonics of uncertain mixtures and dosages to alleviate common ailments. Not all participants had confidence in herbalists, but they are cheap and accessible compared to modern medicine.

In Nasarawa, men who “practice these conservative religions” reportedly did not allow their wives to go to hospitals because they might be under the care of male health providers.

“Some of my friends will tell you that … their husbands will not allow them to go to hospital, especially these people that practice these conservative religions. They believe that when they (the wives) go to hospitals, men (male health providers) will attend to them, so they don’t want men to “look” at them, understand? So they don’t want men to “look” at their wives. These are some of the reasons why traditions affect some people”.

### Husband’s role – from men’s perspective

FGDs with husbands in Nasarawa State corroborated the range of attitudes and behaviours that facilitate or present barriers to uptake. Many discussions referenced cost of drugs and of transportation to access care that husbands were expected to cover.

“It depends on the money for that medicine, even if it is one million naira provided you have the money, you just pay”.

Husbands also considered it their duty to ensure that their wives take prescription medication. It was implicit in many of the discussions that there is a belief that women in Nasarawa needed to be “forced”, or husbands needed to “make sure” women “went out” to seek health care. There were also suggestions that some husbands threaten physical violence against their wives to ensure that the women seek care for protection against malaria and comply with the prescriptions.

“Yes, you have to, because you have to force her before she takes the medication”.

Some husbands mentioned other kinds of support provided for their pregnant wives, such as assisting with household chores and ensuring they eat foods thought to be healthy.

***“****You know whenever a woman becomes pregnant, there are two to three things which the husband is supposed to be doing for her… Difficult work must be stopped, because anything that will touch her and what she is carrying inside her stomach; secondly she must take additional supplements”.*

“Encouragement—you have to encourage her in one way or the other, maybe you have to pay for the transport money to take her, make sure you remind her of the appointment even she forgot to take her drugs regularly”.

Apart from encouraging, advising and providing financial support, there was ample evidence from the discussions to support the belief that men constitute an extremely important support mechanism for women seeking medical care during pregnancy. The tone of both male and female discussions suggested that if a man wants his wife to seek medical care in a health facility, the woman virtually has no other alternative. Male and female participants variously portrayed men as the initiator, financier, advisor and enforcer of hospital attendance and compliance with prescriptions by women.

“I will make sure I collect the card for her then I will leave her with the doctors; I have done my part.

“The help is that we force them to come for antenatal”.

## Second level of influence: community

Extended family members and peers often influence the choice of place of treatment. Respondents reported receiving support and encouragement from some family members such as their parents or a trusted neighbor or friend when they seek health care during pregnancy. Some women might not go to a clinic unless such a person encouraged them to do so. Friends or neighbors might pressure pregnant women not to skip appointments and to seek medical help when ill.

“A friend came to visit me. She saw how terribly ill I was. After two days, she came to visit me and she saw my condition had not improved. Therefore, she told me to seek medical attention or she will never visit me again. So, to me she was caring; that was why she advised me”.

Women also reported that friends shared good experiences about medical care and instigated companionable trips to medical facilities.

“If my friend wants to go to the hospital, she normally calls me and we go together”.

Other women attending ANC facilities provided significant collective support.

“Each time we come for antenatal care, we make new friends. Sometimes we talk about the facilities and the drugs; sometimes we encourage ourselves to take our drugs. A woman may say ah! They gave me malaria drug and I have not taken it, and another woman may say why have you not been taking it? Somebody might say I have not been taking my traditional herbs, and another person will say try to take them because they will help you. We encourage one another”.

As with husbands, some parents do not encourage their daughters to go to a hospital during pregnancy because of their traditional beliefs and recommend traditional birth attendants, herbalists or native doctors rather than health care providers in government health facilities.

## Third level of influence: environment

In order to understand the barriers to seeking modern health care against MiP, respondents were asked why some other women in similar conditions would not visit a health facility or why they themselves might hesitate to go to a health facility.

### Women’s perspective

#### Economic barriers to seeking MiP care

Accessibility of services was not a major problem, although distance to a secondary or tertiary health facility is longer and transportation is slightly more expensive in the peri-urban areas relative to the rural location. Though Cross River State should provide free primary health care for pregnant women and children under five, this is not the case in some facilities. Participants in men’s and women’s groups reported that poverty is a major barrier to seeking care at health facilities. Some participants argued that caught between hunger and illness, some people will chose food over anti-malarial drugs. This emphasizes the depths of poverty faced by many in this area.

“Some of our husbands have no money to give us; some are very poor, and one cannot go hospital, even government hospitals, without money. So your only option is to stay at home with malaria and have faith in God to cure you”.

“You see the money for this antenatal is not something big, but no matter how small an amount is, if you don’t have it, it is big”!

“Some women, because they cannot afford the good food they were instructed to eat in the hospital, they do not come back”.

“We know the situation in Nigeria now. Our people most of them – 90 percent—cannot eat good food, three square meals; they are after food, not malaria medicine! Food”!

When they cannot afford to go to the health facility, women would go to local drug shops (patent medicine vendors) for alternative or cheaper malaria drugs of possibly lower quality.

#### Non-economic barriers to seeking MiP care

Among non-monetary reasons identified by FGDs as barriers to uptake of MiP services, rude health providers and long waiting times were noted most frequently. Patients get frustrated by delays they encounter at the public health facilities. Women generally waited for less than two hours in most primary health facilities, but waits could be as long as four hours at the secondary and tertiary health facilities. Many rural participants did not consider transportation to be a problem, but those in peri-urban areas complained about the difficulty of getting to a tertiary facility and the time taken by the trip. Some of them have to travel long distances to get to the facility only to be told that they have arrived too late in the day and cannot be attended to.

“You may get to the clinic and be sent back and asked to return another day. You may lose patience and go into the bush to cut the medicinal leaves to treat yourself with”.

Participants expressed trust in public health care facilities and providers, yet some also complained about negative provider attitudes. Some participants expressed a reluctance to attend services and face provider disapproval if they were not complying with instructions about nutrition and self-care, or were using traditional medicines.

“Some (health staff) you will ask a question, they will be nagging at you”.

#### Incentives for seeking MiP care

Both women and providers mentioned incentives for women to seek health care, or to return for a second IPTp dose. One powerful incentive is a positive experience of effective care, as described by this participant:

“They gave me medicine; after I took the medicine I became well. That motivates me to come for the second time”.

Women and providers described drugs and other commodities such as LLINs as motivating women to seek ANC.

“Sometimes, some women came to my house to inform me that they were given net and drugs free by the health providers, so it motivates me to go to the hospital”.

“We give them delivery packs, some supplied by the Governor’s wife, called ‘mama kits’. They contain two gloves, razor blade; suction tube, two tablets of baby soap, cord clamp, and a baby receiver to motivate then to keep coming to the facility”.

However, the practice of giving material incentives has its own problems, particularly when many clinics have problems maintaining drug supplies.

“Like these nets we give to them is a kind of motivation to them. When you don’t give them these nets, when they come to the clinic most of them will say, I will not come to your clinic again; I will go to another clinic where they will give me nets”.

#### Provider perspective

Health providers at smaller facilities said they might see one to four cases of MiP per day, while those at secondary and tertiary facilities might see as many as 30 per week. Providers said that facilities charge for services. User fees at secondary and tertiary facilities ranged widely, from N350–2,000 (approximately USD2.20–12.60) in both States.

#### Provider knowledge of MiP and IPTp protocols

Community health workers generally knew more about linking ANC to IPTp than facility-based providers. Most health care providers interviewed correctly identified signs and symptoms of malaria, including headache, fever, loss of appetite, chills, general body weakness, vomiting, and abdominal discomfort. Nearly all agreed that malaria is dangerous for a pregnant woman and her foetus. Providers in Cross River seemed more knowledgeable about the effects of malaria on mother and foetus than their counterparts in Nasarawa.

According to the national protocol for IPTp, pregnant women may receive IPT with SP administered as directly observed therapy (DOT) regardless of symptoms, from 16 weeks of pregnancy for the first dose and one month later for the second dose. Pregnant women who have malaria may receive SP or another approved artemisinin-based combination therapy (ACT). All providers interviewed identified two or more anti-malarial drugs, including SP, ACT such as Coartem, and quinine. However, nearly all of them mentioned one or two incorrect drugs, including cholorquine, which has been discontinued and withdrawn from the market due to resistance. Despite its ban, many Nigerians are still using chloroquine [20]. Most listed ACT (or Coartem) as a preventive drug for pregnant women, though it is mainly used for treatment.

“We treat according to their gestation. In the first trimester of pregnancy we are taught to use quinine tablets. At the second trimester you give ACT and then report any cases of complicated malaria to a higher level of care”.

When asked to state how they administer SP for IPTp, none mentioned DOT or other approved steps. When asked directly if IPTp was administered by direct observation or during home visits, the reply was often negative. One noted that the practice of home visits had been discontinued.

“We did it before seriously, because then we had some free drugs to give them, but for now there is no more free drugs. Even though we go to their place, they always ask us, “You only come to make noise; you will not give us anything”?

Incorrect information on malaria case management and dosages was conveyed during interviews.

“Presently it has been prescribed that these ACT be given to pregnant women. Sometimes the administration of the drug differs based on the condition of the woman. For example, you can take the three tablets, which are for a start. For some women, they are given two tablets to start and one to complete the dose”.

No provider interviewed mentioned obtaining rapid diagnostic tests. This suggests that malaria diagnosis is still presumptive based on non-clinical presentations. Health care providers do not appear to have received adequate training in correct dosage and case management of MiP. Many provider statements suggest that focused training is needed to improve malaria case management in health facilities.

#### Provider training

Providers indicated they had attended training on at least one of the following areas: use of rapid diagnostic tests for malaria, IPTp with SP, malaria case management, and interpersonal communication. Though malaria prevention and control is a component of ANC, provider interviews suggest that this is not reflected in the training offered to providers. Many respondents said they heard of focused antenatal care (FANC) and knew of people who had been trained in this area, but few had been trained in it themselves. No provider in Nasarawa who had attended training on malaria prevention and case management had attended any training on FANC. Though one midwife said she had attended FANC training two years earlier, most had not attended such training, and several had not heard of FANC. Participants expressed considerable interest in attending FANC training.

“Focused antenatal care: I have not heard that one, but if something like that can be organized I will attend. There are probably some new trends in the treatment of malaria in pregnant women that I need to be aware of which I am not, and probably this is a new programme, probably different from the training I have obtained in the past, and then any other workshop that will be of use to me as a midwife, that will help me and improve my own service to the pregnant woman”.

While a few practitioners reported they had attended workshops led by experts on new modalities, many providers, especially in Nasarawa, said they had not received any recent training on MiP. Some referred to workshops and seminars they had attended, but most providers said they needed more training.

“Trainings in these areas are necessary and should be more often to update knowledge and boost treatment therapies. I have never had a training concerning malaria. We need training like workshops, capacity building, and seminars on malaria prevention in pregnant women”.

#### Stock-outs

One of the components of the IPTp protocol is DOT, but DOT is not possible if the drugs are not available on site. In cases of stock-outs, providers write prescriptions, but they are unable to tell if the patient filled the prescription or took the medication correctly. Some participants were also unsure if they were adhering to the doctor’s instructions.

“At times if drugs are not available in the health facility, they do write (prescriptions) and give to us to go and buy in the chemist or pharmacy, but we don’t understand what they write”.

Providers in nearly all facilities in Nasarawa said they prescribed drugs that patients purchased outside the facility, mainly because of stock-outs. Providers reported that sometimes SP and other commodities might be freely available at their facilities, but ACT might be out of stock for two or three months. According to one of the physicians interviewed, such stock-outs were due to “the government’s lack of political will”.

Providers in only one health facility in Cross River reported that most of the required malaria commodities were available. (Some providers in large facilities were not sure, since these facilities keep drugs in pharmacies or stores.) Providers also indicated that malaria commodities were available only when supplied by international development partners.

While Cross River health facilities can sometimes alleviate supply problems by buying essential drugs on the open market and charging patients, facilities in Nasarawa either could not afford to buy drugs or their clients could not afford to buy drugs from them. As one midwife put it:

“No, we don’t have them. It’s only before, in the last year, [the government] brought us SP. As they gave us free, we give them free. And here in the community, if you buy something and ask them for the money, they will be complaining. So I don’t need to buy. I only write [a prescription] for them”.

When asked to estimate the proportion of their clients who purchase the anti-malarial drugs that are prescribed, providers said they did not know, and it depended on whether they could afford them.

## Discussion

Results of a published review [18] of 37 qualitative studies on social and cultural factors affecting uptake of MiP interventions in Africa align with results from this study. The review found similarly low levels of awareness and knowledge of IPTp services among pregnant women and the general population. Many women do not clearly understand the difference between prevention and treatment of malaria, and may be reluctant to take medication for a sickness they do not have. When they are sick, a range of factors determines whether pregnant women go to formal health facilities or traditional practitioners for treatment, or self-treat. However, women who seek care at facilities trust their providers and tend to accept medications their providers give them to prevent or treat malaria. Other institutional factors such as lack of supplies limit the implementation of IPTp policies.

### Facilitators of IPTp uptake

#### Individual and community

Most FGD participants demonstrated relatively good knowledge of malaria signs and symptoms and preventive methods, and nearly all knew that mosquitoes cause malaria. Many participants also reported causes of malaria other than mosquitoes, but some of these are simply misunderstandings of health information, and result in the correct preventive behaviour. For example, dirty surroundings will not cause malaria, but keeping one’s compound clean, without standing water, may reduce the population of mosquitoes breeding.

The sample was mostly recruited from women who had attended ANC services, so an overall positive view of ANC was expected and found, even among those who sometimes complained about specific aspects of ANC. Some women reported attending many more than the recommended four visits. Many of the studies in the systematic review [18] reported fear of side effects of SP including miscarriage or difficult deliveries as a result of babies growing too big. FGD participants in this study did not seem to fear that SP might harm the foetus; potential side effects described affected the woman, and were more unpleasant than dangerous.

Husbands are especially concerned about their wives’ health when they are pregnant, and anxious for them to comply with recommended treatment. In general, husbands, other relatives and community members support ANC, IPTp and malaria treatment for pregnant women, and encourage the women to attend. Women in the community seemed to motivate one another to seek routine care as well as treatment.

#### Environment

According to Ministry of Health policy, ANC includes malaria prevention and treatment, and there is a nationwide protocol for IPTp with SP. It is often not followed, particularly because of stock-outs and providers’ lack of specific knowledge, but the policy framework for IPTp is in place, and providers have basic knowledge and desire to learn more.

#### Behavioural and environmental barriers to IPTp uptake

Table 4 summarizes the barriers to IPTp uptake found at each of the three levels of the conceptual framework. One star (*) indicates that a barrier may exist but does not have a very strong influence of lowering IPTp uptake. It may have been mentioned but only by a minority of participants, or participants did not consider it to be very important, or it may not be likely to influence IPTp in particular. For example, most women had a good understanding of the cause and symptoms of malaria, but there were also some misconceptions about causes. These barriers are relatively low priority. Two stars (**) indicate barrier considered to have a more important influence on IPTp uptake. Three stars (***) indicate a serious barrier to IPTp uptake that urgently needs to be addressed. The absence of stars indicates that a barrier of that type did not exist at that level**.** Table 4 summarizes and ranks the barriers to IPTp uptake identified in this study.

**Table 4:** Summary of barriers to intermittent preventive therapy in pregnancy uptake identified in study

**Behavioural and environmental barriers to IPTp uptake**	**Individual and family**	**Community**	**Environment**
**Knowledge and beliefs of cause and symptoms of malaria**	*	*	—
**Knowledge and beliefs of risks of MiP**	**	**	—
**Understanding of IPT**	***	***	—
**Husband’s/community’s support for accessing routine ANC**	*	*	—
**Husband’s/community’s support for accessing MiP care**	**	**	__
**Costs of seeking care**	*	—	*
**Waiting time**	—	—	**
**Provider attitudes**	—	—	**
**Provider knowledge of MiP**	—	—	**
**Provider training in MiP and focused ANC**	—	—	**
**Provider knowledge of IPTp protocols**	—	—	***
**Availability of SP on-site**	—	—	***

(*) indicates that barrier exists that has minimal influence to lower IPTp uptake.

(**) indicate barrier has important influence on IPTp uptake.

(***) indicate serious barrier to IPTp uptake that urgently needs to be addressed.

#### Individual and community

Most women in this study attended the number of ANC visits recommended or more, yet due to stock-outs they still did not receive two doses of IPTp. If the subset of women who are attending multiple ANC visits are not getting complete IPTp, it may mean that the problem resides at the health system or facility level.

Many women lack specific knowledge or have different perception of risks of MiP and the difference between prevention and treatment, and why prevention is necessary. Many FGD participants did not have correct information on medications that treat and prevent malaria, or on IPTp timing and dosage, even if they had previously received IPTp or malaria treatment while pregnant. Some considered traditional medicine as the appropriate first-line treatment for malaria.

The support of husbands and, to a lesser degree, extended family and friends, affects whether and where women seek care. When family and friends have differing perceptions of risk (severity and susceptibility) and the utility of IPTp, they can make it difficult for women to seek care. Some husbands and older family members indicate that other factors associated with ANC visits include cost, preference for traditional medicine, or because they see pregnancy as a normal occurrence that does not necessary require medical intervention.

For many families, lack of financial resources is a barrier, even if they are in favour of ANC. Even if ANC services are free, there are often costs associated with additional tests or services, prescriptions if there is no medication in stock, and transport. Inability to pay may prompt pregnant women who have malaria to seek treatment from traditional providers or other cheaper sources. However, this barrier is not specific to IPTp; rather, it is more likely to prevent women from accessing ANC services at all than to prevent them from receiving IPTp as part of ANC.

#### Environmental

Providers need and want more training in early diagnosis, MiP case management and IPTp-SP, including dosage, spacing, timing, and DOT. ANC providers lacked the necessary knowledge to provide the recommended prevention and treatment interventions for MiP, or to correctly inform women about them. Community health workers tended to be better at linking IPTp to ANC than providers in facilities. Provision of more MiP training and post-training follow-up for ANC front-line workers, particularly facility-based providers, is essential for full implementation of the Nigerian government’s tripartite approach to MiP: use of LLINs, administration of IPTp-SP, and improved case management.

Many facilities selected for the study do not appear to offer focused ANC. Instead, women may attend many more visits without receiving the key elements of the focused ANC package, including IPTp. State malaria control programmes and federal reproductive health programmes need to coordinate to harmonize the ANC and malaria training work plans.

Some facilities, particularly secondary and tertiary level facilities, are located far away for rural communities and have very long waits, which discourages some women from attending ANC if they do not feel unwell. Similarly, some women may avoid care if they expect to be treated disrespectfully, particularly if they have not complied with previous advice.

The biggest barrier to receiving IPTp identified was the frequent stock-outs in facilities. In order for DOT to work, the facility must have the drug available on site. Even those women who do leave and fill a prescription at a local drug store or patent medicine vendor are not likely to come back to take the medication in front of the provider or to report that they took the medicine (nor would this be a desirable addition burden to place on the women). Thus, providers have no way of keeping track of who has taken IPTp and who still needs it, or who is taking it correctly. Given the low levels of specific knowledge about doses and timing among women, this is a particular problem.

#### Opportunities to increase IPTp uptake

The purpose of this study was to identify opportunities to improve uptake of IPTp. Information from women, husbands and providers agrees that a large proportion of the obstacles are structural and require remedies at the health system level, particularly improved supply chain management to reduce stock-outs, and better training and post training follow-up and supportive supervision for providers. A study of IPTp provision in Enugu state [21] using provider interviews and observations also found low levels of provider knowledge and adherence to IPTp. The authors concluded that the main barriers to IPTp were at the provider and facility level, rather than at the community level [22] and recommended improved training along with supervision and monitoring. Table 5 presents steps that programmes can take to improve MiP and particularly IPTp uptake, at each of the three levels.

**Table 5:** Opportunities for intervention to improve intermittent preventive therapy in pregnancy uptake

	**Level of intervention**		
**Barrier to IPTp uptake**	**Individual**	**Community**	**Environment**
Understanding of MiP	Emphasize the importance and benefits of timely treatment of malaria with ACT, rather than herbal medicines to women and their families.	Emphasize the importance and benefits of timely treatment of malaria with ACT, rather than herbal medicines to women and their families.	Develop and disseminate effective job-aids for providers on focused ANC and MiP protocols.
			Partner with CHWs to reach and inform women and their families on ACT.
Understanding of IPT	Develop lower literacy information packets to explains dosage and timing for women given prescription for SP.	Develop lower literacy information packets to explains dosage and timing for women given prescription for SP.	Provide supportive health care supervision to ensure that providers and CHWs follow MiP protocols.
Costs of seeking care	Emphasize lower cost of preventing malaria compared to treating malaria or complications (such as low birth weight infant)	Emphasize lower cost of preventing malaria compared to treating malaria or complications (such as low birth weight infant)	Ensure that only allowable fees are charged.
Waiting time	Identify and promote specific day of the week as “malaria treatment day” to ensure attendance and speedy services delivery.	Identify and promote specific day of the week as “malaria treatment day” to ensure attendance and speedy services delivery.	Provide appropriate staffing to ensure timely services delivery on “malaria treatment day” and reduce waiting time.
Client-provider interaction	Encourage women seeking MiP services to ask questions and interact with providers.	Include messages encouraging women to ask providers questions in BCC campaigns	Improve provider IPC skills through short, intensive training with role plays to enable providers to see patient’s perspectives.
Availability of SP on-site	NA	NA	Improve supply-chain management. May require a more inclusive facility-level and mobile drug distribution processes.
Women, Families and Community	Encourage women and families to see benefits rather than barriers to preventive MiP care seeking behaviours.	Reinforce existing beliefs and behaviours to prevent and manage MiP	Engage influential community members to encourage prevention and management of MiP

Communication programmes can support training initiatives and improve provider competence in a number of ways. Job aids can help providers counsel women on prevention and treatment of MiP, and prescribe IPTp correctly. Providers are often reluctant to use job aids for fear of seeming to lack knowledge, but a patient brochure that can be used for counselling and given to the pregnant woman to take home may be more acceptable. Such a brochure could use clear pictorial instructions to explain how the woman should take IPTp, which is particularly important if most women have to buy SP from a pharmacy, and when she should return for her next dose. Women’s trust of providers affects their willingness to take unfamiliar medicines. Training providers on interpersonal communication and providing services in a respectful manner may increase the likelihood that women comply with their instructions to take SP, even if they find it unpleasant.

Community-level activities to reduce barriers or reinforce existing beliefs and behaviours that facilitate appropriate prevention and management of MiP must target not only pregnant women but also their husbands, families and influential community members. Though FGD participants indicated they had received information about malaria during ANC visits and from community health workers, they also had misconceptions about causes, signs and symptoms and particularly the risks of MiP. Studies have shown that when pregnant women do not perceive MiP as a serious illness, or they have misconceptions about its causes, they may be less willing to comply with recommended methods of prevention, such as IPTp or use of ITNs, or treatment with ACT [18]. Husbands play important roles in whether their wives access health care, but FGD participants in Cross River expressed a willingness to go against their husband’s wishes if necessary to benefit the health of their baby. Women and men suggested that husbands are more supportive of their wives’ health care when it protects the foetus. Community health workers (CHW) are trusted sources of information, and may effectively promote IPTp and prompt use of ACT for MiP in the context of protecting the foetus from the risks of malaria.

At their September 2012 meeting, the Malaria Policy Advisory Committee to the WHO decided to recommend that IPTp-SP protocols be changed from two doses to four doses [23]. One dose is to be given at each focused ANC visit, at least one month apart, beginning in the second trimester and up to delivery. In this context, it is more urgent than ever to address the barriers to the uptake of IPTp among pregnant women who are attending ANC.

#### Limitations of study

The biggest limitation of this study is that it used purposive sampling and recruited primarily from among women who were already accessing services, and so does not adequately capture barriers for women who may not be attending ANC. However, the primary interest of the study was to learn more about the MiP care that women receive, which can be best accomplished by speaking to women who are already accessing care. The issues preventing women from accessing ANC may be very different from those preventing ANC patients from receiving IPTp. While only one-third of women receiving ANC are also receiving IPTp, there is a clear need to increase the quality of MiP care provided, as distinct from increasing the number who are accessing it.

An additional limitation relates to stock-outs. At the time of the study, many facilities had high levels of stock-outs. This problem may have been resolved post-study. Further, the existence of frequent stock-outs may have influenced provider adherence to protocols or even provider experience. For example, providers may rely on pharmacies to explain to women how to take the medication. Areas with greater drug security may not experience some of the barriers that we identified.

## Abbreviations

ACT: Artemisinin-based combination therapy; ANC: Antenatal care; CHW: Community health workers; DOT: Directly observed therapy; FANC: Focused antenatal care; FGD: Focus group discussion; IDI: In-depth interviews; IPTp: Intermittent preventive therapy in pregnancy; ITN: Insecticide-treated bed net; LGA: Local government areas; LLIN: Long-lasting insecticide-treated nets; MiP: Malaria in pregnancy; NGO: Non-government organization; SEM: Socio-ecological model; SP: Sulphadoxine-pyrimethamine; WHO: World Health Organization.

## Competing interests

The authors declare that they have no competing interests.

## Authors’ contributions

CD and TP conceived the paper, designed the analysis and wrote the first draft of the manuscript. CM contributed substantially to the analysis and writing. CM and BK contributed to editing the manuscript. All authors have read and approved the final version of this paper.
